# Graphene-based terahertz bias-driven negative-conductivity metasurface

**DOI:** 10.1039/d2na00288d

**Published:** 2022-07-01

**Authors:** Guibin Li, Guocui Wang, Tingting Yang, Yan Zhang, Jingling Shen, Bo Zhang†

**Affiliations:** Key Laboratory of Terahertz Optoelectronics, Ministry of Education, Advanced Innovation Center for Imaging Technology, Beijing Key Laboratory for Terahertz Spectroscopy and Imaging, Beijing Key Laboratory of Metamaterials and Devices, Department of Physics, Capital Normal University Beijing 100048 China bzhang@cnu.edu.cn yzhang@cnu.edu.cn

## Abstract

A graphene-based terahertz negative-conductivity metasurface based on two types of unit cell structures is investigated under the control of an external bias voltage. Electrical characterization is conducted and verification is performed using a finite-difference time-domain (FDTD) and an optical-pump terahertz (THz)-probe system in terms of simulation and transient response analysis. Owing to the metal-like properties of graphene, a strong interaction between the metasurface and monolayer graphene yields a short-circuit effect, which considerably weakens the intensity of the resonance mode under passive conditions. Under active conditions, graphene, as an active load, actively induces a negative-conductivity effect, which enhances the THz transmission and recovers the resonance intensity gradually because of the weakening of the short-circuit effect. The resulting resonance frequency shows a blue shift. This study provides a reference value for combining graphene exhibiting the terahertz bias-driven negative-conductivity effect with metasurfaces and its corresponding applications in the future.

## Abstract 2

A graphene-based terahertz negative-conductivity metasurface based on two types of unit cell structures is investigated under the control of an external bias voltage.

## Graphical abstract

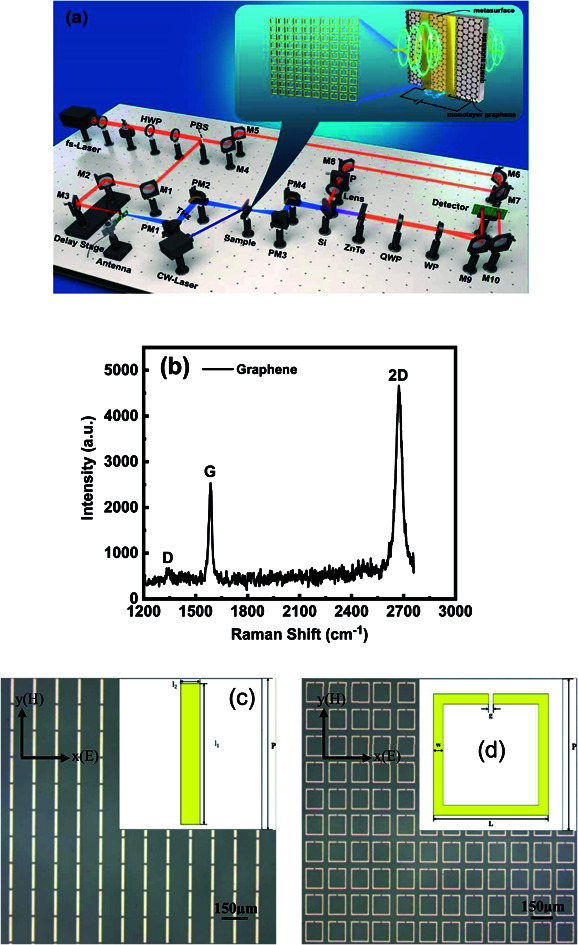

## 1. Introduction

Terahertz (THz) waves are electromagnetic waves between the microwave and infrared bands in the electromagnetic spectrum, with a frequency range of 0.1–10 THz. THz waves avoid the application risks associated with high-frequency waves (such as X-rays) with high energy and strong destructive force, solve the problem of severe Rayleigh scattering in intermediate frequency waves (such as visible light and near-infrared light) with short wavelengths, and compensate for the low spatial resolution attributed to long microwave wavelengths. On the basis of these factors, THz waves are widely used in remote sensing, homeland security, astronomy, nonlinear applications, material diagnostics, broadband wireless communications, and many other fields.^1–5^

Metamaterials are a group of artificial materials with a regular array of periodic subwavelength structures composed of metal or dielectric materials in the entire space area. When resonant metamaterials interact with incident electromagnetic fields, they show electromagnetic response characteristics not possessed by natural materials. These characteristics include a negative refractive index, artificial magnetism, and superfocusing to achieve the flexible control of the incident light phase, amplitude, and polarization.^6–11^ Compared with fully three-dimensional metamaterial structures, metasurfaces occupy less physical space, thus offering the possibility of low-loss structures.^6^ Because resonant metasurfaces can interact with the incident electromagnetic field, obvious resonant absorption can be realized in the THz frequency range by adjusting the parameters of a metasurface unit cell, such as its period, linewidth, gap width, and medium.^12–14^ However, once the parameters of metasurface unit cells are determined, the THz resonance absorption becomes stable and unchangeable, considerably limiting the applications of metasurfaces. Therefore, metasurfaces are typically combined with other materials to form a hybrid metasurface for achieving the flexible control of THz waves by applying external control conditions to the device.^15–18^

Graphene is a single-atom form of carbon with a sp^2^-hybridization honeycomb crystal plane. Since its first successful preparation in 2004,^19^ monolayer graphene has shown high carrier mobility, broadband flat optical response, tunable bandgaps, high electronic and optical conductivities, and many other unique electrical and optical properties, thus garnering extensive research attention.^20–23^ The conductivity of graphene can be effectively regulated *via* chemical doping or by applying externally excited light or an external bias voltage, possibly enabling the combination of graphene and metasurfaces.^24–26^

In this work, a graphene-based terahertz negative-conductivity metasurface device is proposed in which the unit cell structure of the metasurface is designed as a rectangular split-ring resonator (RSRR) and a rectangular metal-rod resonator (RMRR). Without an external bias voltage, a very weak resonance peak of the metal dipole appears under THz transmission. When an external bias voltage is applied to both electrodes of the samples (*i.e.*, RMRR/graphene/quartz and RSRR/graphene/quartz) with increasing applied voltage, the THz transmission gradually increases, the intensity of the resonance peak of the metal dipole is gradually recovered, and the resonance frequency shows a blue shift. Therefore, the stable control of the device is realized under an external bias voltage. The conductivity of the device is calculated using the equivalent transmission line theory for elucidating the enhancement in THz transmission. A finite-difference time-domain (FDTD) and an optical-pump THz-probe system are used to verify the abovementioned experimental results.

## 2. Experimental details

**Fig. 1:**
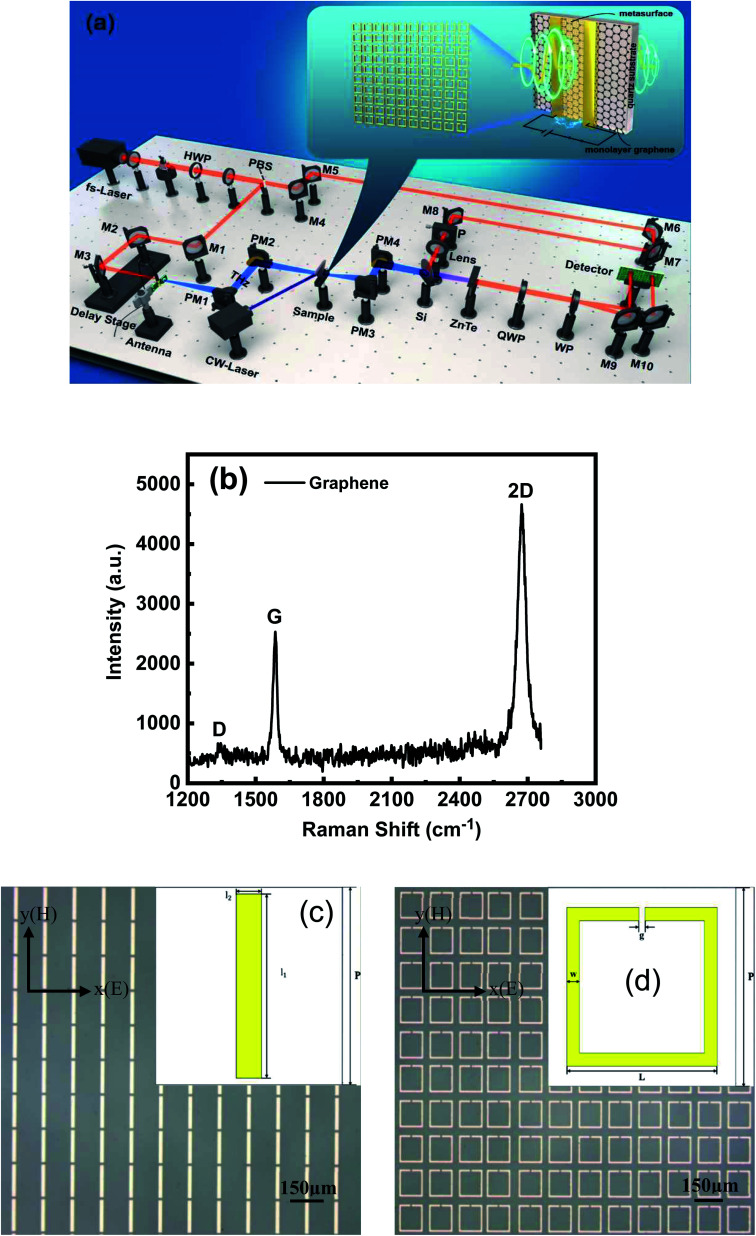
(a) Schematic of the terahertz time-domain spectroscopic (THz-TDS) system and the sample used in this study. (b) Raman spectrum of the monolayer graphene on a quartz substrate. Microscopic images of the (c) rectangular metal-rod resonator (RMRR) and (d) rectangular split-ring resonator (RSRR). Illustrations show the structural parameters of the unit cell. Scale bar = 150 μm.

The experimental measurement system is a THz time-domain spectroscopic (THz-TDS) system (Fig. 1(a)). The enlarged image in Fig. 1(a) shows a schematic of the sample structure and metasurface. First, monolayer graphene sheets were fabricated at low and high temperatures *via* chemical vapor deposition and transferred to a clean quartz substrate with dimensions of 2 cm × 2 cm × 1 mm. Fig. 1(b) shows the Raman spectrum of the monolayer graphene on a quartz substrate. The locations of the G peak at ∼1586 cm^−1^, the 2D peak at ∼2673 cm^−1^ and the almost disappeared D peak all indicate the high quality of monolayer graphene. Second, the metasurface was formed at the center of the monolayer graphene sheet *via* ultraviolet lithography. The metasurface is gold film with a thickness of 200 nm. Microscopic images of the two metasurfaces with different designs are presented in Fig. 1(b) and (c), where the structures and parameters of the unit cell are shown. The structural parameters of the RMRRs are as follows: length *l*_1_ = 140 μm, width *l*_2_ = 20 μm, and period *P* = 150 μm. The structural parameters of the RSRRs are as follows: outer length *L* = 120 μm, linewidth *W* = 10 μm, gap width (functioning as an equivalent capacitor) *g* = 5 μm, and period *P* = 150 μm. Finally, the center line of the sample is considered as the symmetrical axis and two parallel gold electrodes with a length, width, thickness, and spacing of 2 cm, 5 mm, 200 nm, and 3 mm, respectively, were plated on both sides of the center line of the sample *via* vacuum thermal evaporation. Thus, the complete sample structure required for the experiment was prepared.

## 3. Results and discussion

**Fig. 2:**
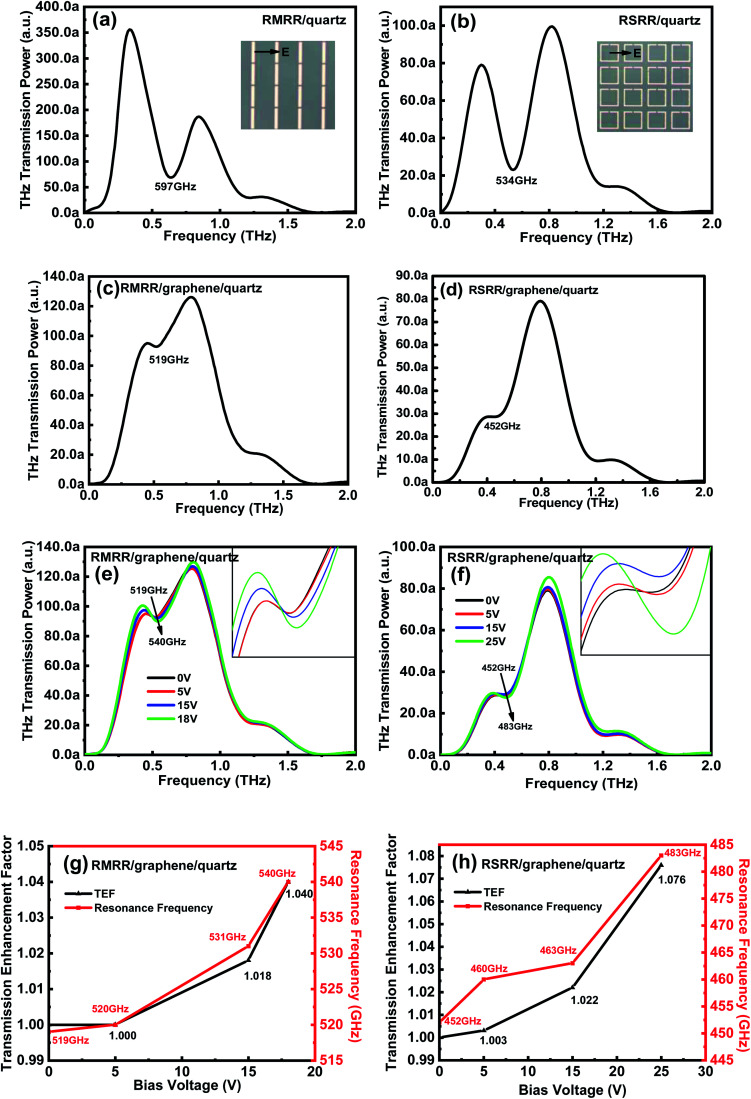
THz transmission frequency-domain signals of (a) RMRR/quartz, (b) RSRR/quartz, (c) RMRR/graphene/quartz, and (d) RSRR/graphene/quartz samples without an external bias voltage. THz transmission frequency-domain signals of (e) RMRR/graphene/quartz and (f) RSRR/graphene/quartz samples with different external bias voltages. The amplification section highlights the variation in resonance absorption. The dependence of the transmission enhancement factor and the resonance frequency on the bias voltage of (g) RMRR/graphene/quartz and (h) RSRR/graphene/quartz.

The RMRR/quartz and RSRR/quartz samples demonstrate obvious resonance absorption at 597 and 534 GHz, respectively (Fig. 2(a) and (b), respectively). The experimental results show that the samples without monolayer graphene exhibit a bright dipole resonance mode with high intensity and a broad linewidth. The RMRR/graphene/quartz sample shows weak resonance absorption at 519 GHz. Moreover, the RSRR/graphene/quartz sample exhibits weak resonance absorption at 452 GHz (Fig. 2(c) and (d)). With the addition of monolayer graphene, the intensity of the dipole resonance mode of the two types of metasurface samples (*i.e.*, the RMRR/graphene/quartz and RSRR/graphene/quartz samples) decreases considerably and the resonance frequency shows a substantial red shift. An external bias voltage is applied to the electrodes of the RSRR/graphene/quartz and RMRR/graphene/quartz samples. With increasing external bias voltage, the THz transmission gradually increases, the resonance intensity is recovered, and the resonance frequency undergoes a certain blue shift. The resonance frequency of the RMRR/graphene/quartz metasurface sample undergoes a blue shift from 519 to 540 GHz (Fig. 2(e)). The resonance frequency of the RSRR/graphene/quartz metasurface sample shows a blue shift from 452 to 483 GHz (Fig. 2(f)). In order to quantitatively analyze the transmission enhancement phenomenon of the terahertz wave, the transmission enhancement factor (TEF) is defined as follows1
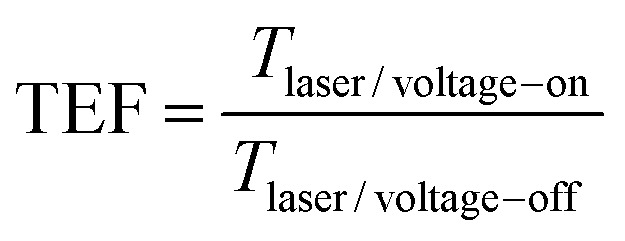
Where *T*_laser/voltage−on_ represents the integral of the frequency-domain spectrum intensity of the transmitted terahertz wave when the bias voltage is applied, and *T*_laser/voltage−off_ represents the integral of the frequency-domain spectrum intensity of the transmitted terahertz wave when no bias voltage is applied. The dependence of the transmission enhancement factor and the resonance frequency on the bias voltage of the RMRR/graphene/quartz and RSRR/graphene/quartz samples was obtained by calculation and analysis, and the corresponding TEF values and the resonance frequency at each bias voltage have been marked (Fig. 2(g) and (h), respectively). In general, the transmission enhancement factor and the resonance frequency have a good monotonic dependence on the bias voltage.

The results show that the resonance of the two types of metasurface resonators corresponds to the dipole resonance mode in the absence of monolayer graphene. The dipole resonance mode is a type of bright-mode resonance excited by a dipole-like parallel surface current in the resonator. The dipole resonance mode is almost independent of the unit cell symmetry and exists in both symmetric and asymmetric split-ring resonators. Since the dipole resonance mode is a bright-mode resonance with strong radiative coupling to the free space, it has high resonance intensity and broad resonance linewidth.^27^ With the addition of monolayer graphene, the dipole resonance mode of the RSRR/graphene/quartz and RMRR/graphene/quartz devices changes considerably. The resonance intensity clearly decreases, and the resonance frequency undergoes a large red shift owing to the strong interaction between the metasurface and monolayer graphene. Alternatively, the metasurface generates a strong electric field in the capacitance gap of the resonator by limiting the electric field to high subwavelength numbers, thus increasing the interaction strength between the field and monolayer graphene.^27,28^ Moreover, the position of monolayer graphene in the device is crucial. Compared with the graphene/metasurface/quartz structures, a stronger interaction is observed between the metasurface in metasurface/graphene/quartz structures and monolayer graphene because of their better electrical contact.^28^ These two factors induce considerable changes in the resonance intensity and resonance frequency of the dipole resonance mode before and after the addition of monolayer graphene.

**Fig. 3:**
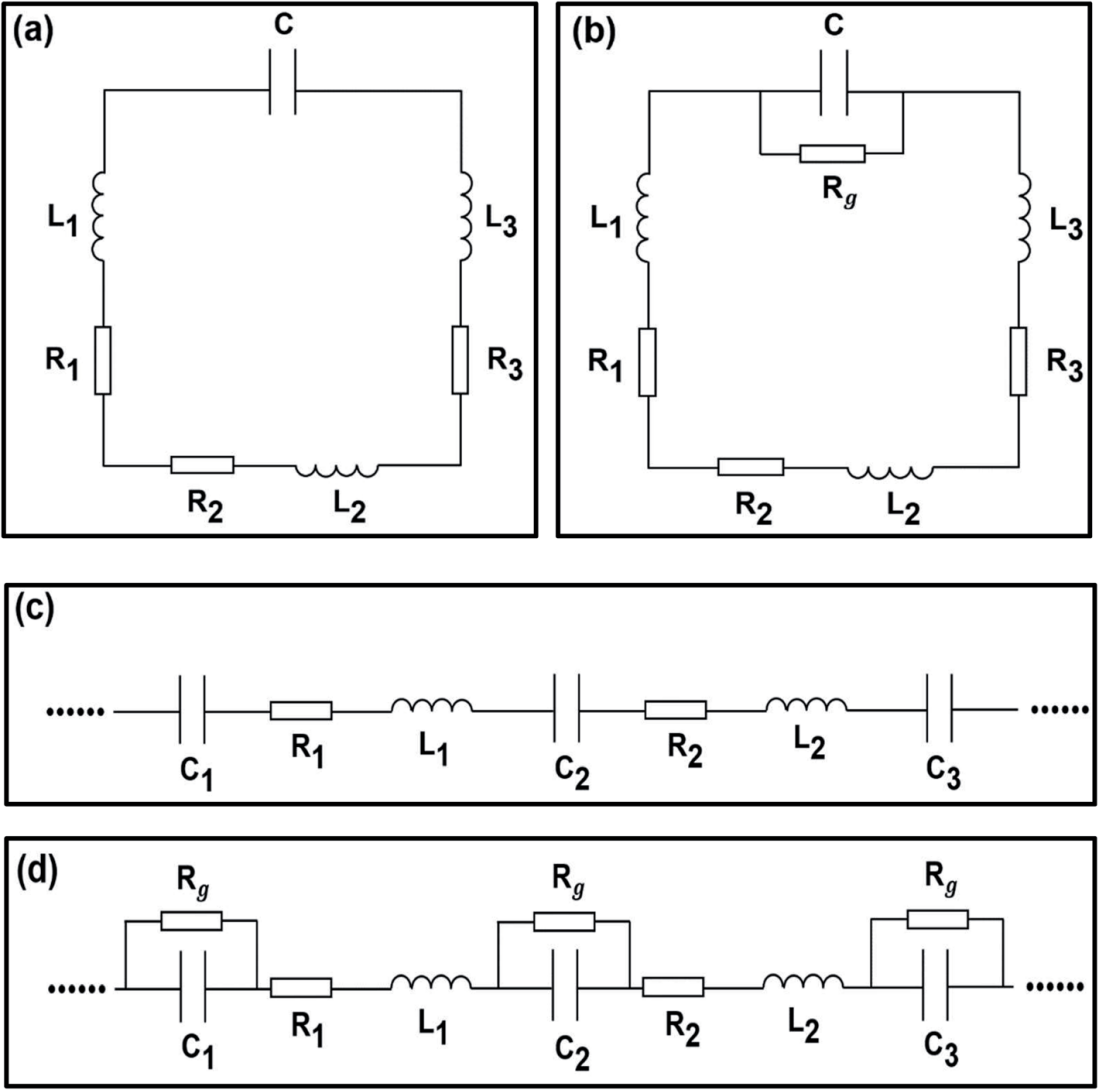
Equivalent circuit models of (a) RSRR/quartz, (b) RSRR/graphene/quartz, (c) RMRR/quartz, and (d) RMRR/graphene/quartz.

To further clarify the interaction mechanism between the metasurface and monolayer graphene, an equivalent circuit model is employed.^27,29–31^ The metallic strip of the resonator provides inductance *L*, the dissipation provides resistance *R*, and the gap provides capacitance *C* (Fig. 3(a)–(d)). Fig. 3(a) and (c) presents the equivalent circuit models of the RSRR and RMRR without monolayer graphene, respectively. Because of the charge-accumulation effect, considerable charge accumulation is observed at both ends of the narrow gap of the resonator, namely, at both ends of the equivalent capacitance, inducing a strong electric field in the resonator gap; this observation corresponds to the dipole resonance mode with high intensity and broad linewidth. When monolayer graphene is added to the sample, because of the metal-like properties of graphene, a small parallel resistance exists at both ends of the equivalent capacitance (Fig. 3(b) and (d)). This phenomenon induces the short-circuit effect, which considerably suppresses the electric field in the capacitance gap and greatly reduces the strength of the electric field, thus severely decreasing the intensity of the dipole resonance mode.

**Fig. 4:**
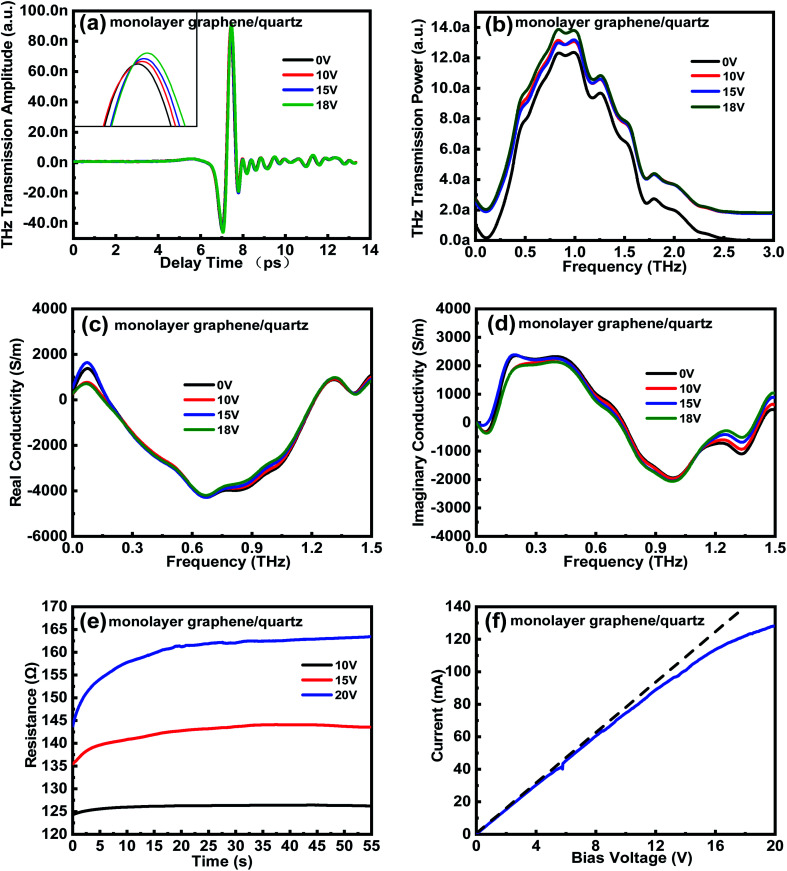
THz (a) time-domain spectra and (b) frequency-domain signals of the graphene/quartz substrate. Conductivities of (c) real and (d) imaginary part of the graphene/quartz substrate calculated using the equivalent transmission line theory before and after applying the external bias voltage, respectively. (e) Resistance–time (*R*–*T*) and (f) current–voltage (*I*–*V*) curves of the graphene/quartz sample.

When the external bias voltage is applied at the electrodes of each sample, the THz transmission improves substantially (Fig. 4(a) and (b)). To explain this phenomenon, the electrical properties of the samples with graphene/quartz structures are characterized. The equivalent transmission line theory is used to calculate the change in sample conductivity. The complex transmittance function of conductive films can be expressed as^32,33^2
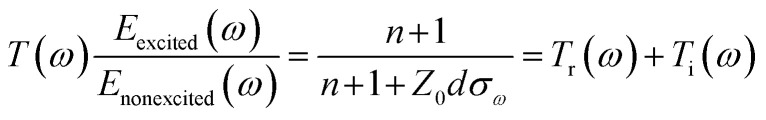


The real and imaginary parts of the film conductivity are then obtained as follows:3
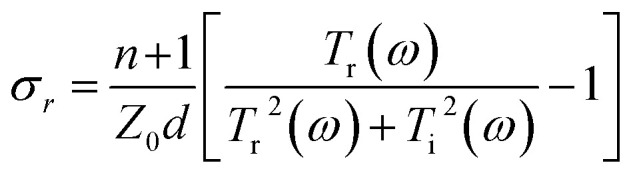
4
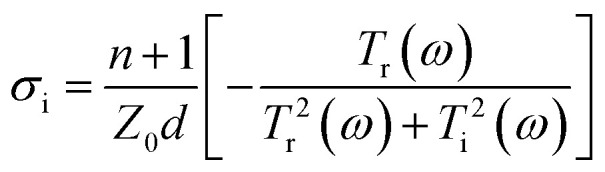
where *E*_excited_(*ω*) and *E*_nonexcited_(*ω*) denote the electric field intensities of the THz waves passing through the samples with and without the application of external control conditions, respectively. Moreover, *d* denotes the sample thickness, *ω* represents the angular frequency of the THz waves, *Z*_0_ denotes the free-space impedance, and *n* represents the refractive index of the quartz substrate. The complex conductivity of the sample calculated using the equivalent transmission line theory is shown in Fig. 4(c) and (d).

The conductivity of both the real and imaginary parts of the monolayer graphene film decreases with an increase in the external bias voltage. The THz transmission is enhanced by the decrease in the conductivity of the monolayer graphene film. Consider the change in conductivity is Δ*σ* = *σ* − *σ*_0_, where *σ* and *σ*_0_ represent the complex conductivities of the sample with and without the application of external control conditions, respectively. Then, the aforementioned results show that when an external bias voltage is applied to the sample with monolayer graphene/quartz structures, Δ*σ* < 0, corresponding to the negative-conductivity effect.^34,35^ Therefore, the negative-conductivity effect of graphene enhances the THz transmission under the application of an external bias voltage to the samples.

To validate the variation trend of the sample conductivity calculated using the equivalent transmission line theory, Keithley's 2450 semiconductor test system is used to evaluate the graphene/quartz sample and the resistance–time (*R*–*T*) and current–voltage (*I*–*V*) curves of the sample are obtained (Fig. 4(e) and (f), respectively). Fig. 4(e) shows the *R*–*T* curve of the graphene/quartz sample under different external bias voltages. The resistance of the monolayer graphene film gradually increases (*i.e.*, the conductivity gradually decreases) until saturation is reached, and this phenomenon becomes increasingly prominent with increasing external bias voltage. Based on Fig. 4(f), the slope of the curve gradually decreases with increasing external bias voltage, implying that the conductivity of the monolayer graphene film gradually decreases. Therefore, the *R*–*T* and *I*–*V* curves of the graphene/quartz sample are highly consistent with the variation trend of the sample conductivity obtained using the equivalent transmission line theory.

Under the applied external bias voltage, in addition to the obvious enhancement in THz transmission, the intensity of the dipole resonance mode is gradually recovered and the resonance frequency undergoes a certain blue shift. Therefore, for the equivalent circuit models of the two metasurface samples (*i.e.*, the RSRR/graphene/quartz and RMRR/graphene/quartz samples), the resistance *R*_g_ in parallel with the equivalent capacitance *C* increases. As *R*_g_ increases, the inhibition effect of monolayer graphene on the electric field in the capacitance gap decreases; thus, the short-circuit effect is weakened and the intensity of the dipole resonance mode is recovered. Alternatively, under the applied external bias voltage, numerous carriers are injected into the device, affecting the dipole resonance mode and inducing a blue shift of the resonance frequency.

**Fig. 5:**
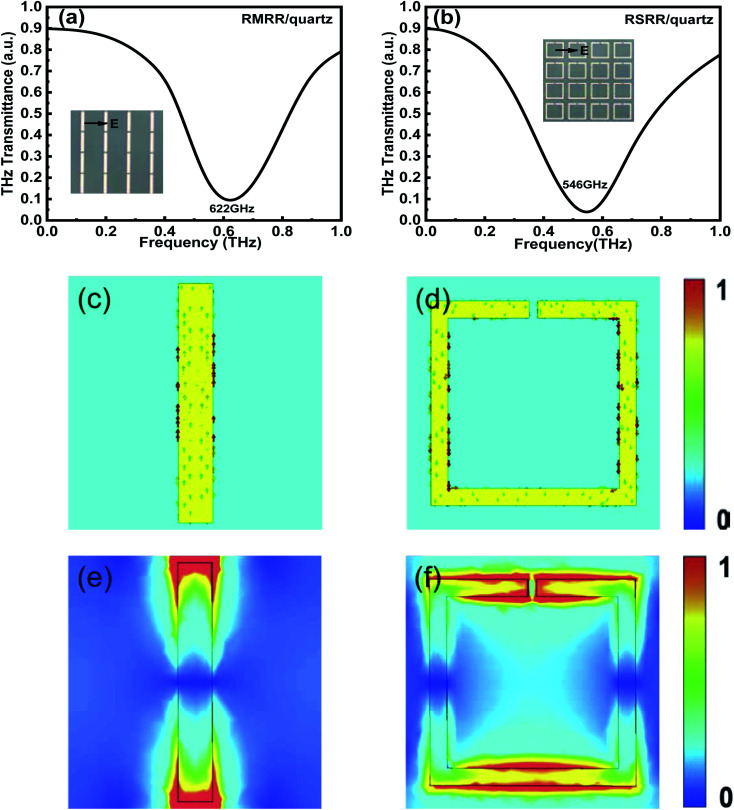
Simulation results of the THz frequency-domain signals of (a) RMRR/quartz and (b) RSRR/quartz metasurface samples, (c) surface current and (e) electric field distribution of the RMRR unit cell structure, and (d) surface current and (f) electric field distribution of the RSRR unit cell structure.

To prove the reliability and validity of the aforementioned experimental results, a finite-difference time-domain (FDTD) solver is used for simulation verification. Fig. 5(a) and (b) shows the simulated frequency-domain signals of the two types of metasurface resonators without monolayer graphene. The resonance frequencies of the RMRR/quartz and RSRR/quartz metasurface samples are detected at 622 and 546 GHz, respectively (Fig. 5(a) and (b), respectively). The resonance of both these samples corresponds to a bright dipole resonance mode with high intensity and broad linewidth, which is consistent with the experimental results. Fig. 5(c) and (e) shows the surface current and electric field distribution of the RMRR/quartz metasurface sample, respectively. The surface current graph reveals that the free electrons in the RMRR flow from one end of the rod to the other end, causing the accumulation of heterogeneous charges at both ends of the RMRR, inducing the entire RMRR to form into a dipole-like structure. Thus, the excited resonance corresponds to the dipole resonance. The electric field distribution graph reveals that a strong electric field is distributed at both ends of the RMRR owing to the accumulation of numerous heterogeneous charges at both ends of the resonator. Therefore, the resonance excited by the RMRR metasurface sample without monolayer graphene corresponds to a bright dipole resonance mode with high intensity and broad linewidth. Similarly, Fig. 5(d) and (f) shows the surface current and electric field distribution of the RSRR/quartz metasurface sample, respectively. Based on the surface current graph, the free electrons in the RSRR start moving from the metal arm opposite to the capacitive gap, form two parallel currents through the left and right parallel metal arms, and finally accumulate on both sides of the capacitive gap of the resonator. Consequently, the entire RSRR forms two dipole-like structures; thus, the excited resonance also corresponds to the dipole resonance. The electric field distribution graph reveals a strong electric field distribution at both sides of the capacitive gap of the RSRR and on the metal arm opposite to the capacitive gap, caused by the accumulation of numerous homogeneous charges on both sides of the gap and the accumulation of several heterogeneous charges between both sides of the gap and the metal arm opposite to the gap. Therefore, the resonance excited by the RSRR metasurface sample without monolayer graphene is also a bright dipole resonance mode with high intensity and broad linewidth. The resonance modes of the RMRR and RSRR are essentially the same: both are dipole resonance modes. Furthermore, for both types of metasurface resonators without monolayer graphene, a strong electric field distribution is observed at the corresponding capacitive gap of the resonator, which explains the strong interaction between the metasurface and monolayer graphene.

**Fig. 6:**
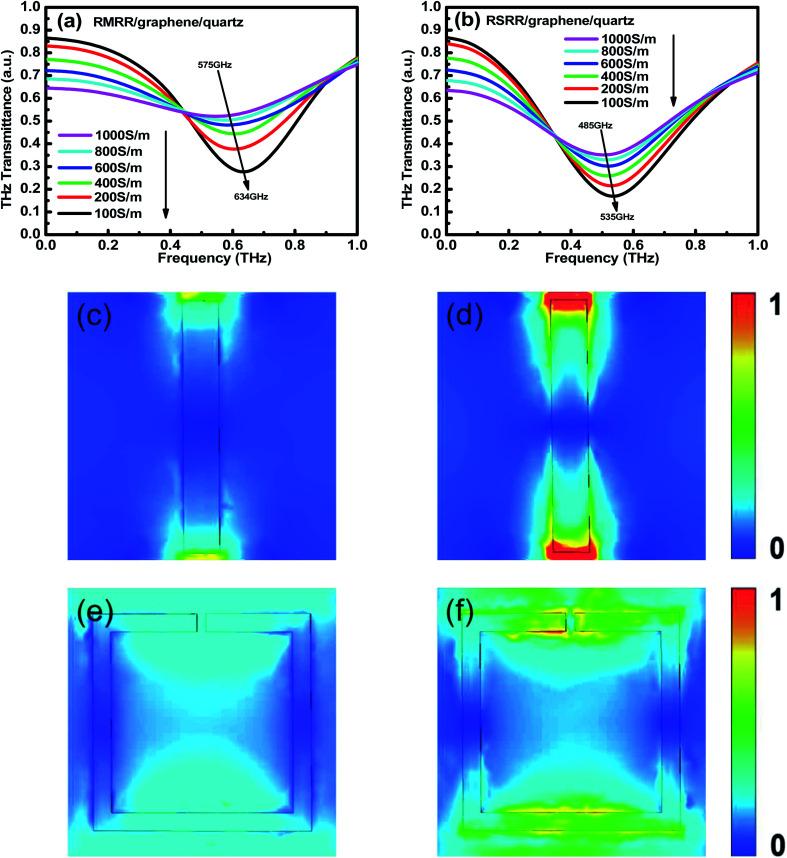
Simulation results of THz frequency-domain signals of (a) RMRR/graphene/quartz and (b) RSRR/graphene/quartz metasurface samples with different graphene conductivities, electric field distributions of RMRR unit cell structures with graphene conductivities of (c) 1000 S m^−1^ and (d) 100 S m^−1^, and electric field distributions of RSRR unit cell structures with graphene conductivities of (e) 1000 S m^−1^ and (f) 100 S m^−1^.

For the two types of metasurface resonators with monolayer graphene, the conductivity of monolayer graphene decreases in the simulation using finite-difference time-domain to a value equivalent to that obtained when applying an external bias voltage in the experiment (Fig. 6(a) and (b)). Compared with the case without monolayer graphene, the resonance frequency of the metasurface/graphene/quartz sample in the simulation results undergoes an obvious red shift. Moreover, its resonance intensity decreases considerably. The conductivity of monolayer graphene gradually decreases from 1000 to 100 S m^−1^. With decreasing conductivity of monolayer graphene, the resonance intensity is gradually recovered and the resonance frequency undergoes a blue shift. Clearly, the resonance frequency of the RMRR metasurface sample shows a blue shift from ∼575 to ∼634 GHz and that of the RSRR metasurface sample shows a blue shift from ∼485 to ∼535 GHz. Notably, as the conductivity of monolayer graphene decreases, the resonance intensity in the simulation results is gradually recovered but ultimately does not recover to that of the sample without monolayer graphene; these results are consistent with the experimental results.

Fig. 6(c) and (d) shows the electric field distribution of the RMRR unit cell structures when the conductivities of monolayer graphene are 1000 and 100 S m^−1^, respectively. At 1000 S m^−1^, the field strength at the capacitance gap of the adjacent RMRR is very weak. Alternatively, at 100 S m^−1^, the field strength at the capacitance gap improves considerably. This explains the gradual recovery of the resonance intensity of the dipole resonance mode with decreasing conductivity of monolayer graphene. Similarly, Fig. 6(e) and (f) shows the electric field distribution of the RSRR unit cell structures when the conductivities of monolayer graphene are 1000 and 100 S m^−1^, respectively. At 1000 S m^−1^, the field strength at the capacitance gap and on the metal arm opposite to the capacitance gap of the RSRR is very weak. However, at 100 S m^−1^, the field strength at the capacitance gap and on the metal arm opposite to the capacitance gap increases substantially. This also explains the gradual recovery of the resonance intensity of the dipole resonance mode with decreasing conductivity of monolayer graphene.

**Fig. 7:**
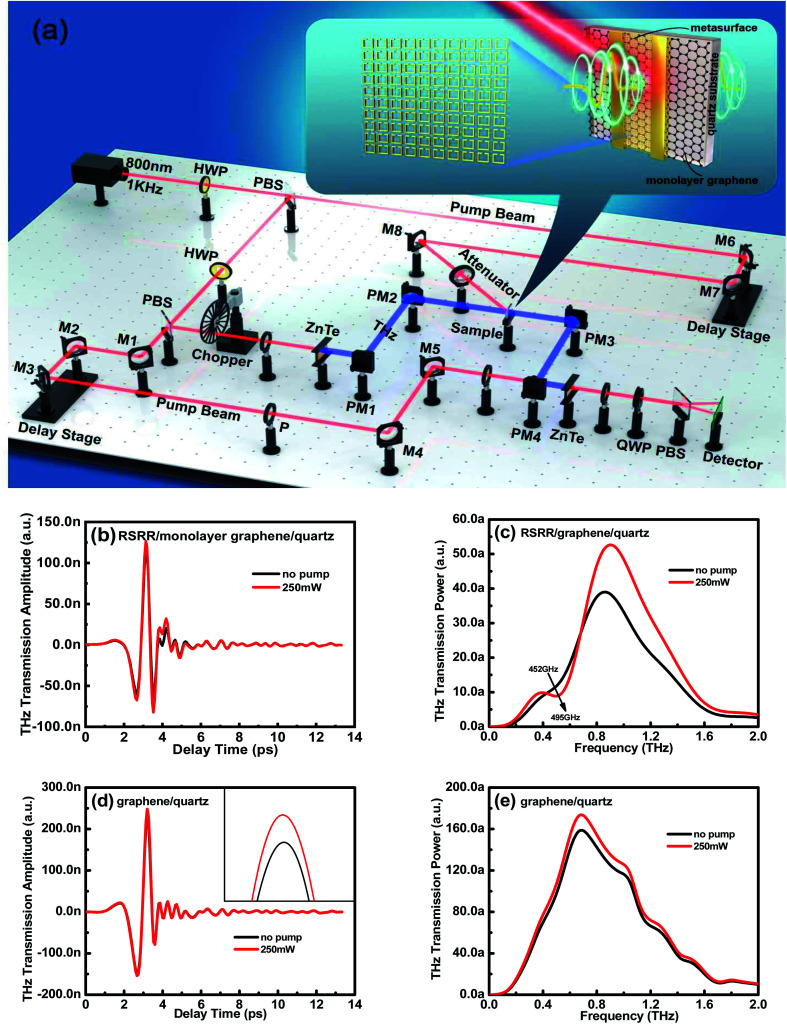
(a) Schematic of the optical-pump THz-probe system and the sample used in this study. THz (b) time-domain spectra and (c) frequency-domain signals of the RSRR/monolayer graphene/quartz sample before and after the application of pump light. THz (d) time-domain spectra and (e) frequency-domain signals of the monolayer graphene/quartz sample before and after the application of pump light.

The optical-pump THz-probe system is used to evaluate the transient response of the samples. Fig. 7(a) shows a schematic of the optical-pump THz-probe system that generates pulses with a repetition rate of 1 kHz at a central wavelength of 800 nm. These pump pulses pass through the chopper, facilitating the detection of THz transmission alternately with and without pump light excitations (*T* and *T*_0_, respectively), thus confirming the pump-induced change in THz transmission Δ*T*_r_ = *T* − *T*_0_. The enlarged image in Fig. 7(a) shows a schematic of the sample used in this study. First, the transient response of the RSRR/graphene/quartz sample is evaluated using the optical-pump THz-probe system. When the sample is excited using pump light, the THz transmission is clearly enhanced, resonance intensity is recovered, and resonance frequency undergoes a blue shift from 452 to 495 GHz, consistent with the experimental results obtained by the stable control of the sample with an external bias voltage (Fig. 7(b) and (c)). The transient response of the graphene/quartz sample is estimated using the optical-pump THz-probe system. When the sample is excited using pump light, the THz transmission is considerably improved (Fig. 7(d) and (e)). Thus, the test results for the optical-pump THz-probe system agree well with those obtained *via* the stable control and electrical characterization. Since the conductivity of graphene can be changed by bias voltage or photoexcitation, the proposed structure can be used to fabricate active resonance tunable terahertz metasurface switches, which will enrich the design and application of terahertz functional devices.

## 4. Conclusion

Graphene-based terahertz negative-conductivity metasurfaces with two types of unit cell structures—RSRR and RMRR—under the control of an external bias voltage are investigated. With the addition of monolayer graphene, the resonance intensity of the metasurface decreases considerably and the resonance frequency shows an obvious red shift. When an external bias voltage is applied to the sample, the THz transmission increases gradually, resonance intensity increases gradually, and resonance frequency undergoes a blue shift. The physical mechanism of THz transmission enhancement for the actively induced negative-conductivity effect of graphene is elucidated *via* the electrical characterization of the graphene/quartz samples. A finite-difference time-domain and an optical-pump THz-probe system are used to verify the negative-conductivity metasurface. The combination of graphene's negative-conductivity effect and metasurfaces is expected to promote the further development of THz active hybrid metasurface devices based on graphene.

## Author contributions

The manuscript was written through contributions of all authors. All authors have given approval to the final version of the manuscript.

## Conflicts of interest

The authors declare no conflict of interest.

## Supplementary Material
